# Investigation on the Effect of Thermal and Mechanical Treatment to the Offshore Corrosion Behavior of 6351 Aluminum Alloy in Red Sea Environments

**DOI:** 10.1155/2020/8826366

**Published:** 2020-09-26

**Authors:** Yahya Ali Fageehi, Rajasekaran Saminathan

**Affiliations:** Department of Mechanical Engineering, College of Engineering, Jazan University, Jizan, Saudi Arabia

## Abstract

This study investigates the effect of artificial aging treatment and mechanical attrition treatment on the corrosion behavior of 6351 Al alloy in Red Sea environment. The artificial aging of the alloy is carried out at temperature range 140°C–240°C in steps of 20°C for various time periods after the solution heat treatment at 530°C for 1 hour. Based on the hardness measurements, the aged specimens are categorized into three, namely, underaged, peak aged, and overaged. The as received alloy specimens are subjected to mechanical attrition treatment in a vacuum chamber using steel balls. Vickers hardness test reveals that there is a remarkable improvement in hardness of mechanical attrition treated specimens compared to that of aged specimens. The aged and mechanical attrition treated specimens were subjected to the corrosion test in Red Sea water using the Autolab instrument. The corrosion tests reveal that the peak-aged composite corrodes more in Red Sea water when compared to that of other groups of specimens. XRD measurements and SEM analysis are carried out to study the surface nature of attrition treated specimens. It is observed that the mechanical attrition treated specimens exhibit a nanocrystalline surface and lead to a decrease in corrosion resistance. However, the annealing of the alloy after attrition treatment optimizes the mechanical and corrosion properties of the alloy.

## 1. Introduction

Steels are very important class of materials finding applications in marine and submarine industries since they possess very good mechanical properties combined with good formability. Even though they are superior in mechanical properties, they are heavier in nature, and hence, the cost of transportation of goods in marine industries is comparatively higher. There is a need for exploring new light materials of similar mechanical properties in construction domain of marine industries [1–4]. From the past decades, the research is being carried out to find alternate materials for marine applications, and few of the potential candidates are the aluminum alloys possessing good mechanical properties, excellent corrosion resistance, and light in weight [5–10]. Among the promising aluminum alloys, 5xxx and 6xxx alloys are considered to be marine grade since they possess excellent corrosion resistance when compared to that of other aluminum alloy series. The alloy 6351 Al alloy contains magnesium, silicon, and manganese as alloying elements. The considered alloy comes under the heat treatable aluminum alloys category, so that there exists a need for obtaining the appropriate heat treatment cycle in order to achieve desirable mechanical properties [11–14]. During the heat treatment, the formation of alloying elements precipitate in the matrix depending on the time and temperature of the treatment. The precipitates differ in size and shape with respect to the heat treatment conditions, which in turn decide the mechanical properties and corrosion behavior of the matrix in aggressive environments [15–17]. The corrosion behavior of the aluminum alloys in various sea environments are studied since the saline nature, surface temperature, and water circulation pattern of seas in the world are different. This indicates that there is a need to carry out the research in finding the corrosion behavior of the alloys of marine grade at different sea conditions [18–21]. Very meager information are available in literature about the corrosion behavior of aluminum alloys in Red Sea conditions [22]. The surface strength of aluminum alloys in terms of chemical stability has been improved by techniques such as anodization in which anodic oxidation gives rise to a stable oxide layer [23, 24]. Chromating process involving introduction of hexavalent chromium as the alloying element in the surface by chemical processes [25] and rare earth material coating such as CeO_2_ is employed by chemical methods to protect the surface from aggressive chemical attack [26]. Conversely, to obtain the mechanical stability of the alloy surface, addition of hard particles such as boron carbide and graphite to the surface and severe surface mechanical treatments (SSMT) such as shot peening, laser shock peening, and surface mechanical attrition treatment are used to deform the alloy surface to achieve enhancement in surface strength [27–29]. Surface mechanical attrition treatment (SMAT) is one among the efficient techniques to improve the surface strength by refining the grain size leading to nanograins formation [30, 31]. In addition, the severe plastic deforming of materials by various methods leads to grain refinement to the desired level and enhances the mechanical properties without affecting the surface chemical behavior to a large extend [32–37].

The literature clearly suggests that there is a need for exploring new techniques to improve surface strength of aluminum alloys in marine applications. Severe plastic deforming techniques are applicable to improve the surface strength of the alloys. Surface mechanical attrition treatment (SMAT) is one among those available techniques. In addition, the corrosion behavior of the engineering materials is not fully studied in Red Sea environment till now. Hence, there is a scope for carrying out research on the effect of aging and the SMAT process on the chemical and mechanical stabilities of the 6351 Al alloy in Red Sea environments.

## 2. Materials and Methods

### 2.1. Material

In the present work, 6351 Al alloy is under consideration. The material received is in the form of extruded cylindrical rods of diameters 10 cm and 1.5 cm. The composition of the alloy is given in Table 1.

### 2.2. Age Hardening Treatment of 6351 Al Alloys

#### 2.2.1. Solution Heat Treatment

Specimens of 2 cm × 2 cm × 1 cm are cut from the cylindrical rod of 10 cm diameter using a disc cutting machine and subjected to solution heat treatment further. This involves heating the specimens to 530°C for a time period of 1 hour and then followed by quenching in water. All heat treatments are carried out in an induction furnace with a maximum limit of 1000°C with an accuracy of ± 1°C. The holding time of two hours leads to the alloying elements such as Cu, Si, Mg, and Zn go into solid solution and form single-phase solid solution. Overheating and underheating are avoided in the heat treatments carried out. Overheating might result in undesirable grain growth and surface blisters, whereas the underheating might lead to inadequate dissolution of alloying elements in the matrix.

#### 2.2.2. Artificial Aging Treatment

The solution treated specimens were then subjected to artificial aging at temperatures, namely, 140°C, 160°C, 180°C, 200°C, 220°C, and 240°C for a time period of 1 hour to 25 hours. The aged specimens were then polished using emery papers of grade up to 1000 and then cloth polished using diamond paste of 1 micron grade. The polished specimens are then subjected to Vickers hardness testing using a German made Leica model tester with pyramid-shaped diamond indenter. A load of 2 kg is used in testing, and the indentation duration happens to be around 15 seconds. Based on the results obtained in hardness measurements, the specimens are categorized into three namely underaged, overaged, and peak aged. The peak-aged specimen corresponds to the one in which the hardness achieved is maximum when compared to that of other categories.

### 2.3. Surface Mechanical Attrition Treatment (SMAT)

The severe plastic deformation of the surface of the received 6351 Al alloy is achieved by surface mechanical attrition treatment carried out in a vacuum chamber in which the specimen of 2 cm × 2 cm × 1 cm is placed in the sample holder. In this process, steel balls of 5 mm diameter are used as milling media, and the frequency of agitation is set at 40 kHz. The treatment is carried for various time periods from 30 minutes to 240 minutes. The specimens subjected to different time period attrition treatment are further characterized by Vickers hardness measurements. The attrition treated specimen is further subjected to annealing at 300°C for 2 hours to improve the corrosion behavior.

### 2.4. Potentiodynamic Polarization Measurements

The aged and surface mechanical attrition treated specimens are further subjected to potentiodynamic polarization measurements using the Autolab instrument, Metrohm BV model. For electrochemical testing, the aged specimens of 1 cm length and 1.5 cm diameter are chosen since the sample holder of Autolab instrument can accommodate a cylindrical specimen of 1 cm length and 1.5 cm diameter. The attrition treated specimens are also cut into the same dimension for corrosion testing. Except attrition treated specimens, all other specimens were polished with various grades of SiC paper, from 80 up to 1000 grade, and cloth polished using 1 micron diamond paste just prior to immersion in Red Sea water during electrochemical testing. For the electrochemical testing, the geometric area of the specimens exposed was 1 cm^2^. Red Sea water is collected 10 km away from the seashore of Jizan region in Saudi Arabia. 500 mL volume of the electrolyte is used in every electrochemical test. Potentiodynamic polarization scans are employed starting from −1000 mV versus standard calomel electrode and varied to approximately −100 mV, all potentials being relative to the open-circuit corrosion potential. Polarization curves of different specimens were recorded over the range −1000 to −100 mV, and the same are analyzed to obtain corrosion rates. All potentiodynamic scan rates were 0.4 mV/s. Electrochemical cell was composed of a three-neck glass flask, a large area platinum counter electrode, and saturated calomel electrode (SCE) as a reference electrode. All potentials are referenced to the SCE. Polarization measurements give rise to the polarization resistance values of different specimens in electrochemical testing.

The corrosion rate [38] is obtained from the following expression:

(1) $$\begin{array}{c} \text{CR}\left(\text{mpy}\right)=\frac{0.129\times \;I_{\text{CORR }}\times \text{Eq.wt}}{D}, \end{array}$$

where *I*_corr_ is the corrosion current density in *μ*A/cm^2^. Eq. wt is the equivalent weight of the corroding specimen in gm. *D* is the density of the corroding species in g/cm^3^

Corrosion rate may also be presented in millimeter per year.

*I*_corr_ [38] is obtained by dividing the corrosion current (*I*_corr_) value by the area of specimen exposed to the electrolyte.

(2) $$\begin{array}{c} i_{\text{CORR}}=\;\frac{1}{2.3R}\;\left(\frac{\beta_{a}\beta_{c}}{\beta_{a}+\beta_{c}}\right), \end{array}$$

where *R* is the polarization resistance (kΩ/cm^2^), *β*_*a*_ is the anodic slope (volts/decade), and *β*_*c*_ is the cathodic slope (volts/decade).

The topography of the alloy specimens before and after attrition treatment was analyzed using the JEOL JSM-6380LA scanning electron microscope. X-Ray analysis of the aged and attrition treated specimens was carried out using the X-ray diffractometer (JEOL JDX–8P) having the copper (30 kV, 20 mA) target with a *K*_*β*_ filter. The diffracting angles were set to 0°–120° with a scanning speed of 2°/min and in continuous mode.

## 3. Results and Discussion

### 3.1. Artificial Aging Behavior of 6351 Al Alloy

**Figure 1:**
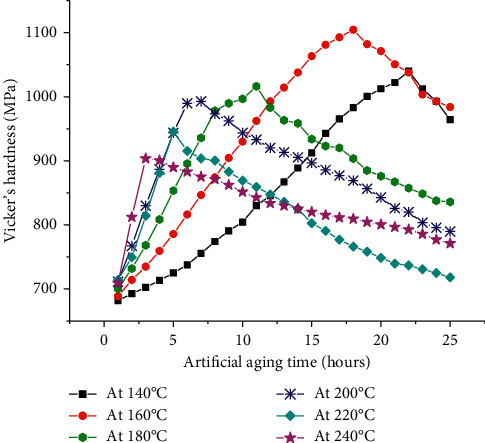
The hardness variations with artificial aging time.

Artificial aging of the heat treatable aluminum alloys are carried out in order to achieve better mechanical properties according to the need of end applications.  Figure 1 shows the artificial aging curves of the 6351 Al alloy drawn between time and hardness. Hardness is expressed in MPa, and the time is scaled in hours. In general, the graph shows the hardness variations with respect to time of artificial aging. Common observation of all the curves in the graph reveals that the hardness increases with aging time and reaching maximum before starting to decrease with respect increase in time. The time to reach maximum hardness differs with variation of aging temperature. At lower temperatures, the time to reach the maximum hardness is higher when compared to that of the same at higher temperatures, namely, 220°C and 240°C. Also, the maximum hardness reached is higher at the lower temperatures, namely, 140°C and 160°C. The proper explanation of the aging behavior of heat treatable aluminum alloys can be elaborated by beginning with the solution heat treatment mechanism. The solution heat treatment at 530°C for a time period of 1 hour leads to the formation of supersaturated solid solution with aluminum as the solvent matrix and Mg, Si, Cu, and Mn as solute atoms. Also, another critical importance of solution heat treatment exists in introducing vacancies in the matrix, and hence leading to the phenomena called self-diffusion. The quenching process in water retains the elemental status of the alloying elements and the introduced vacancies [39].

During artificial aging treatment, at the initial stages, uniformly distributed clusters of Si and Mg atoms form in the supersaturated solid solution (*α* phase). Furthermore, the coclustering of Mg and Si takes place to proceed gradually to form *β* (Mg_2_Si) [40].

The precipitation sequence mentioned by a researcher states that Al-Si-Mg coclusters transform to equiaxed precipitates and then to *β*″ precipitates. The later transform to *β*″ precipitates and then to Mg_2_Si as follows. Separate Mg and Si clusters ⟶ coclusters of Mg and Si ⟶ small equiaxed precipitation ⟶ *β*″ precipitates ⟶ *β*″ precipitates ⟶ *β* (Mg_2_Si).

**Figure 2:**
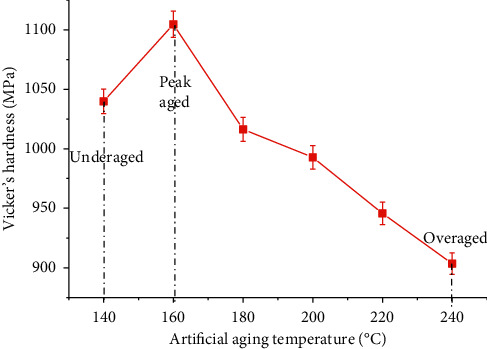
The hardness variations with artificial aging temperature.

In silicon rich alloys, the precipitation of silicon at grain boundaries leads to fracture initiating at the grain boundaries, and this effect is reduced by the addition of Mn in 6351 Al alloy. At lower temperatures, the Guinier–Preston zones (GP zones) transform into needle-shaped precipitates, and at higher temperature, increased size precipitates are observed. The hardness of the alloy depends on the size and density of the precipitates formed [41]. As observed in Figure 2 (the hardness variations with respect to temperature), at a lower temperature range, the maximum hardness achieved is falling into the greater side, whereas at higher temperatures, the maximum hardness values are falling into the smaller side. The specimens of aging time lesser than the time required to achieve peak aging are categorized as underaged, and the specimens of aging time greater than the peak aging time are categorized as overaged.

**Figure 3:**
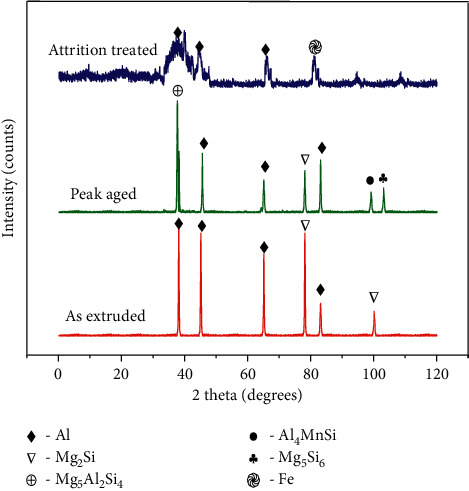
XRD profiles of the 6351 Al alloy.

Figure 3 represents the XRD profiles of as extruded alloy, peak-aged alloy, and attrition treated alloy. The profile corresponding to the extruded sample contains peaks of Mg_2_Si and aluminum. But the peak-aged alloy specimen profile contains peaks corresponding to Mg_2_Si, Mg_5_Al_2_Si_4_, Al_4_MnSi, and Mg_5_Si_6_ precipitates. Hence, the XRD analysis clearly reveals that the magnesium silicide particles transform into multiple precipitates of different composition leading to improvement in hardness and strength of the 6351 Al alloy. The aging response of the 6351 Al alloy is understood from the results obtained as the hardness of the alloy increases with respect to the increase in size of the precipitates. However, when the precipitates become coarser, then the nonuniform distribution of the particles affects the hardness adversely. From the results analysis, it can be clearly stated that the hardness of the peak-aged category is maximum among other categories of specimens, and it can be achieved by carrying out the artificial aging at 160°C for a time period of 19 hours.

### 3.2. Surface Mechanical Attrition Treatment of 6351 Al Alloy

One of the main objectives of the mechanical attrition treatment carried out is to improve the surface hardness of the aluminum alloy, since the aluminum alloys are potential candidates to replace steels in marine and submarine industries [3, 4]. The milling process involves severe plastic deformation of the surface of the alloy by the vibrating steel balls at higher frequencies in random directions.

**Figure 4:**
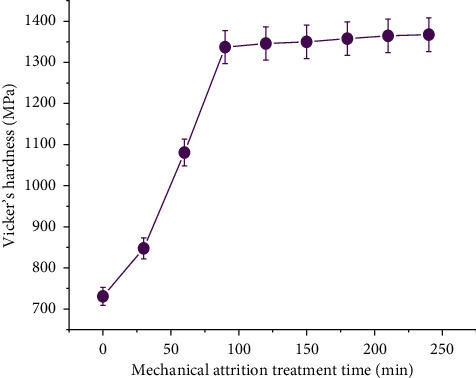
Hardness variation with time of attrition treatment.

The grain refinement in the surface is initiated by the milling process over the surface exposed. The grain refinement continues with time of attrition, and the grains transform from coarser to finer with increase in time of attrition. The micron-sized grains transform into nanograins as time proceeds in attrition treatment [42]. The mechanism of the grain refinement in surface mechanical attrition treatment is quiet complex and explained in terms of dislocation influenced the grain subdivision process [43]. The substrates subjected to surface attrition treatment are divided into two categories, namely, materials with high stacking fault energy (HSFE) and materials with low stacking fault energy (LSFE). Aluminum alloys come under high stacking fault energy materials [43]. During the grain refinement of materials with HSFE, dense dislocation walls (DDWs) and dislocation tangles (DTs) will begin to form well with the grains subjected to grain refinement. Furthermore, these dense dislocation walls and dislocation tangles develop into partial boundaries of misorientation. As the mechanical energy is obtained from the milling process, the partial boundaries yield into complete grain boundaries resulting in grain refinement [43].  Figure 4 is the plot between attrition time and the hardness in MPa. It starts with the lowest hardness corresponding to the as received extruded alloy and proceeds with the hardness values of alloy specimens subjected to attrition treatment for various time periods from 30 minutes to 240 minutes. It is observed that at the initial time period of the attrition treatment, there exists a steep increase in hardness when compared to that of the greater time period attrition treatments. This is related to the fact that, at the initial time period, faster grain refinement takes place since the matrix is transforming from micron level grains to nanograins. Once the nano grains started forming, the matrix structure is having the dual type grains, namely, micrograins and nanograins. As the time progresses, the nanostructured grains are quantified greater in comparison to the micrograins. A stage reaches at which the most part of the matrix has been transformed into nanostructured grains, and hence further transformation takes place through the depth of the matrix. At this time period, hardness variation is gradual as observed in Figure 4. The presence of the nanostructured grains is justified by the XRD profile of attrition treated 6351 Al alloy for 240 minutes as shown in Figure 3. The profile resembles the standard XRD of aluminum nanoparticles obtained from the International Center for Diffraction Data (ICDD).

**Figure 5:**
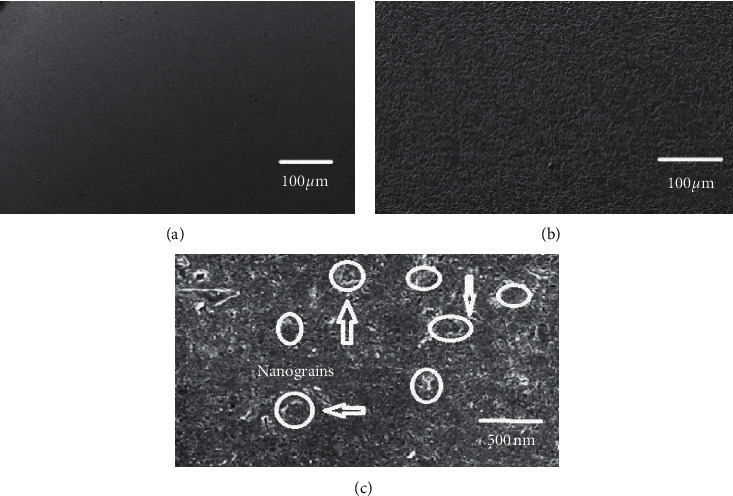
SEM micrographs of 6351 Al alloy (a) as extruded, (b) attrition treated for 240 minutes, and (c) attrition treated for 240 minutes at higher magnification.

Figure 5 shows the micrographs of the alloy before and after attrition treatment.  Figure 5(b) shows that the severe plastic deformation has occurred uniformly throughout the matrix of the alloy.  Figure 5(c) shows that the micrograph at higher magnification clearly reveals that the matrix contains uniformly distributed nanograins. Only the visible grains with grain boundaries are highlighted, and the closer observation reveals that the nanostructured grains are uniformly distributed throughout the matrix. Metallographical analysis proves that nanostructured grains are not aligned in a single direction, and they are oriented in different directions.

### 3.3. Corrosion Behavior of Aged and Attrition Treated Alloy

**Figure 6:**
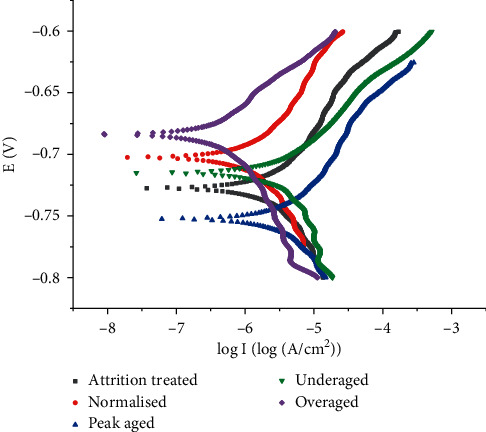
Potentiodynamic polarization plot of 6351 Al alloy in Red Sea water.

Aluminum alloys have better strength and lower corrosion resistance than the pure aluminum due to the addition of alloying elements. The addition of alloying elements disturbs the stability of the inherent oxide layer present over the aluminum. Also, the electrochemical potentials of the alloying elements are differing with respect to that of the aluminum matrix. Even though the considered 6351 Al alloy is of marine grade, the study of corrosion behavior is very essential in order to utilize the same in end applications of various offshore and onshore regions of the world. Also, another important factor affecting the corrosion behavior of the alloy is the heat treatment cycle leading to the formation of precipitates in different sizes and shapes throughout the matrix [17]. This leads to the possibility of galvanic corrosion of the alloy when exposed to aggressive media such as seawater. The surface temperature, water circulation pattern, and saline nature of Red Sea differ with respect to that of the other seawaters of the world [44].  Figure 6 shows the potentiodynamic polarization curves of the 6351 Al alloy subjected to various thermal (solution and aging treatment) and mechanical treatments (SMAT). The corresponding corrosion characteristics, namely, the zero current potential, corrosion current, and corrosion rates of the aged, attrition treated, and normalized 6351Al alloy, are tabulated in Table 2.

The results of the potentiodynamic polarization experiments carried out in Red Sea water (Figure 6) show that the polarization curves of underaged and overaged specimens shift toward the left with higher zero current potential (ZCP) and lower current densities (I_CORR_) compared to the polarization curves of peak-aged specimens. This indicates that there is an increase in corrosion rate for the artificially aged specimens at 160°C for 19 hours (peak aging condition).

**Figure 7:**
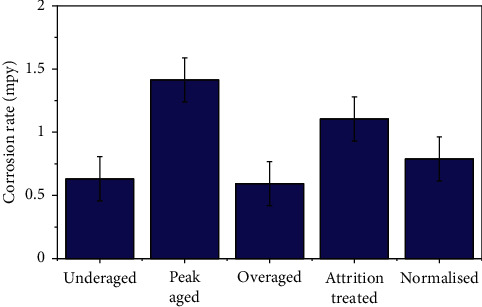
Corrosion rates of 6351 Al alloy in Red Sea water.

Aluminum comes under the category of the extremely reactive metals group. However, the presence of inherent stable oxide layer protects the metal in aggressive conditions. Addition of alloying elements of differing chemical potential to the aluminum matrix during alloy making leads to a decrease in corrosion resistance due to the formation of the precipitates in the matrix, promoting the galvanic corrosion [17]. [15]. The XRD profile (Figure 3) reveals that the peak-aged specimen contains Mg_2_Si, Mg_5_Al_2_Si_4_, Al_4_MnSi, and Mg_5_Si_6_ precipitates. Hence, the higher corrosion rate observed for the peak-aged alloy is justified by the presence of various types of precipitates in the matrix. This is clearly evident when the corrosion current and corrosion potentials of the aged specimens are analyzed (Table 2). Hence, the degree of galvanic corrosion depends on the shape and density of the precipitates formed [17]. Also, the common factor that might lead to the corrosion of the aged composite is the aggressive saline nature of Red Sea water.  Figure 7 shows the corrosion rate variation of the alloy categories with standard deviations. Ten specimens were corrosion tested in every category, and the average values were registered. The attrition treated alloy specimens contain large quantity of nanocrystalline grains in the surface when compared to that of untreated specimen surfaces. Higher the number of grains, larger is the ground boundary regions. Being the higher energy regions where the silicon has the tendency to precipitate, the grain boundary regions of higher dislocation density may lead to higher corrosion rate when compared to the untreated specimen. Figure 3 clearly shows that the existence of iron (Fe) peaks revealing the surface alloying of Fe atoms from milling media to the alloy surface during the attrition treatment. Also, another important factor is that the wearing of the milling media may lead to rough surface, and hence affecting the attrition treatment of the alloy. This is evident by the analysis of the corrosion characteristics of the attrition treated specimens.

Hence, the normalizing treatment of the attrition treated specimen were carried out at 300°C for a time period of two hours leading to normalizing the alloy surface by decreasing the dislocation density in the surface. During normalizing treatment, grain growth is also possible. The polarization curve of normalized alloy after attrition treatment shifts toward the left with higher corrosion potential and lower current density (Figure 6). The surface hardness of normalized alloy happens to be 1289 MPa. When the normalizing treatment is carried out at higher temperatures and for longer duration, the surface hardness value decreases to an appreciable level. It can be stated that the normalizing treatment carried out at 300°C for two hours improves the corrosion resistance of attrition treated alloy without much reduction in the surface hardness.

## 4. Conclusion

The artificial aging studies of 6351 Al alloy reveal that the peak aging is achieved by heat treatment at 160°C for a time period of 19 hours. The peak-aged alloy exhibits increased tendency to corrode in Red Sea water when compared to that of underaged and overaged alloys. The attrition treatment of the alloy results in exceptional increase in surface hardness due to the development of nanostructured grains. The corrosion rate of attrition treated alloy surface falls at slightly higher side due to the presence of nanograins. However, the corrosion resistance of the SMAT-processed alloy is improved by annealing treatment at 300°C for 2 hours without compromising much in the surface strength.

## Figures and Tables

**Table 1:** Composition of 6351 Al alloy [13].

Element	Composition (wt%)

Si	1.04
Fe	0.18
Cu	0.04
Mn	0.69
Mg	0.71
Ti	0.02
Cr	0.013
Al	97.3

**Table 2:** The corrosion characteristics of 6351 Al alloy in Red Sea water.

Material/Condition	*E*_CORR_ (V)	*I*_CORR_ (A/cm^2^)	CR (mpy)

Underaged	−0.719	5.13 × 10^−5^	0.632
Peak aged	−0.754	12.98 × 10^−5^	1.415
Overaged	−0.686	4.93 × 10^−5^	0.593
Attrition treated (AT)	−0.732	10.14 × 10^−5^	1.105
Normalized (NR)	−0.708	7.25 × 10^−5^	0.789

## Data Availability

The data used to support this study are made available from the corresponding author upon request.

## Nomenclature

*E*_CORR_: Corrosion potential
*I*_CORR_: Corrosion current density
CR: Corrosion rate.
